# Silent Threat: Incidental Diagnosis and Surgical Management of a Giant Syphilitic Aortic Aneurysm

**DOI:** 10.1155/carm/1281603

**Published:** 2025-12-29

**Authors:** Henrique Madureira Da Rocha Coutinho, José Xavier López, Juan Luis Muñoz Noblecilla, Esmeralci Ferreira, Joaquim Henrique De Souza Aguiar Coutinho

**Affiliations:** ^1^ Department of Cardiovascular Surgery, UERJ/HUPE, Rio de Janeiro, Brazil; ^2^ Department of Cardiology and Hemodynamics, UERJ/HUPE, Rio de Janeiro, Brazil

**Keywords:** aortic arch aneurysm, cardiovascular syphilis, case report, deep hypothermic circulatory arrest, syphilitic aortitis

## Abstract

**Background:**

Cardiovascular syphilis (CVS) is a rare but severe manifestation of tertiary syphilis, often remaining clinically silent until life‐threatening complications develop. Syphilitic aortitis can lead to aneurysmal degeneration, most commonly involving the ascending aorta and aortic arch. Despite advances in public health, the global resurgence of syphilis has renewed concern about its cardiovascular sequelae and the need for diagnostic vigilance.

**Case Report:**

We describe a 59‐year‐old male with systemic hypertension and a remote smoking history, who was largely asymptomatic except for mild exertional dyspnea (New York Heart Association Class II). During routine preoperative evaluation for noncardiac surgery, imaging revealed a giant aortic arch aneurysm measuring 114 × 94 × 93 mm, with associated dilatation of the ascending and descending aorta. Serologic testing confirmed syphilitic infection, and syphilitic aortitis was identified as the underlying etiology. After the completion of benzathine penicillin therapy, the patient underwent successful replacement of the ascending aorta and entire aortic arch under deep hypothermic circulatory arrest with selective antegrade cerebral perfusion. The supraaortic trunks were reimplanted en bloc using the island technique. Postoperative recovery was complicated by pleural effusion and transient neurological symptoms, both of which were managed conservatively, with full resolution. At two‐month follow‐up, the patient had returned to normal daily activities with preserved functional capacity.

**Conclusion:**

This case illustrates an atypical, arch‐predominant presentation of CVS diagnosed incidentally in the contemporary era. It emphasizes the need to consider infectious etiologies, particularly syphilis, in patients with nonatherosclerotic or unusual aortic aneurysm patterns. Early recognition, appropriate antimicrobial therapy, and timely surgical intervention are essential to prevent catastrophic outcomes. The ongoing resurgence of syphilis underscores the importance of maintaining clinical awareness of this “forgotten disease,” especially in patients with atypical aortic pathology.

## 1. Introduction

Syphilis, a chronic systemic infection caused by *Treponema pallidum*, remains a global health concern despite the widespread availability of effective antibiotic therapy since the mid‐20th century [1]. Known as the “great imitator,” syphilis can involve virtually any organ system, with the tertiary stage associated with destructive lesions affecting the cardiovascular and central nervous systems [2].

Cardiovascular syphilis (CVS) typically develops 10–30 years after inadequately treated or untreated primary infection and most often manifests as syphilitic aortitis, aneurysmal dilatation, aortic valve insufficiency, and coronary ostial stenosis [3, 4]. Historically prevalent, CVS has become uncommon in many developed settings due to routine use of penicillin and improved screening. Nevertheless, recent epidemiological data indicate a resurgence of syphilis, driven in part by socioeconomic vulnerability, HIV coinfection, and delayed diagnosis [5, 6].

The pathophysiology of syphilitic aortitis involves obliterative endarteritis of the vasa vasorum, leading to ischemic medial necrosis, fragmentation of elastic fibers, and progressive weakening of the aortic wall [7]. This process predominantly affects the tubular ascending aorta, often sparing the sinuses of Valsalva, and predisposes patients to fusiform aneurysm formation, aortic regurgitation, and, in advanced cases, rupture [8].

In contemporary practice, degenerative and atherosclerotic etiologies account for the majority of thoracic aortic aneurysms. However, infectious causes—including syphilis—should still be considered, particularly in atypical presentations or in patients without classical atherosclerotic risk factors [9]. Syphilitic aneurysms may remain clinically silent until catastrophic complications occur, such as rupture or dissection [10]. Serological tests can also yield false‐negative results in late disease, further complicating diagnosis [11]. Surgical intervention remains the cornerstone of management for large or symptomatic syphilitic aneurysms, while optimal outcomes depend on controlling the underlying infection and ensuring long‐term surveillance of the remaining aorta [12, 13].

We present a case of a giant ascending and aortic arch aneurysm secondary to syphilitic aortitis, incidentally diagnosed during preoperative evaluation for noncardiac surgery. This report highlights diagnostic challenges, the interplay between syphilitic and degenerative changes in the aortic wall, and key aspects of contemporary surgical and postoperative management.

## 2. Case Report

A 59‐year‐old man with systemic arterial hypertension and a history of cigarette smoking from ages 15 to 19 years (approximately one pack per day, ∼4 pack‐years) was under outpatient follow‐up for low‐risk prostate adenocarcinoma (Gleason 6, ISUP 1). During routine preoperative assessment for urological surgery, he reported mild exertional dyspnea consistent with New York Heart Association (NYHA) functional Class II, prompting further cardiovascular evaluation.

A comprehensive social and infectious risk assessment revealed no history of high‐risk sexual practices, illicit drug use, or prior sexually transmitted infections. He reported a monogamous relationship for more than two decades and no family history of syphilis or congenital infection. These details guided interpretation of the serologic findings, supporting late latent or tertiary syphilis rather than recent exposure.

**Figure 1:**
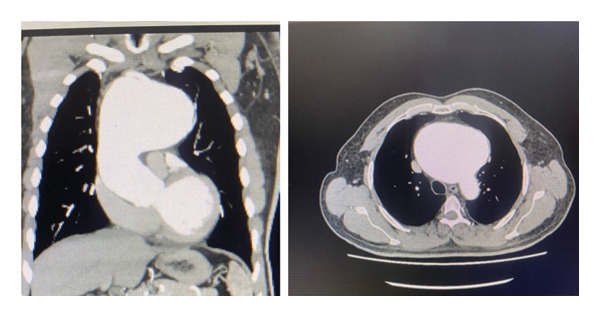
CT angiography images showing a large aneurysm involving the ascending aorta and the aortic arch.

**Figure 2:**
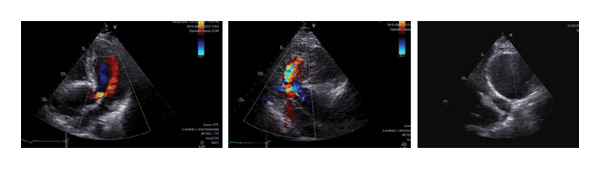
Transthoracic echocardiogram showing aortic regurgitation and aortic dilatation.

Contrast‐enhanced computed tomography (CT) angiography demonstrated a giant aneurysm primarily involving the aortic arch, measuring 114 × 94 × 93 mm, with a longitudinal extension of 79 mm. Additional dilatations were observed in the midascending aorta (49 × 48 mm), distal aortic arch (31 × 28 mm), and proximal descending thoracic aorta (26 × 25 mm) (Figures 1 and 2). The aneurysm morphology and maximal diameter indicated a high risk of rupture, leading to urgent referral for surgical management.

Serologic evaluation confirmed active syphilitic infection, with VDRL 1:32, TPHA positive, and negative HIV screening. The hepatitis panel revealed HBsAg negative, anti‐HBc IgM negative, protective anti‐HBs titers, and anti‐HCV negative, excluding viral hepatitis–related vasculopathy. The patient completed a full course of benzathine penicillin G, 2.4 million units intramuscularly once weekly for three consecutive weeks, consistent with the CDC and WHO recommendations for late latent/tertiary syphilis.

**Figure 3:**
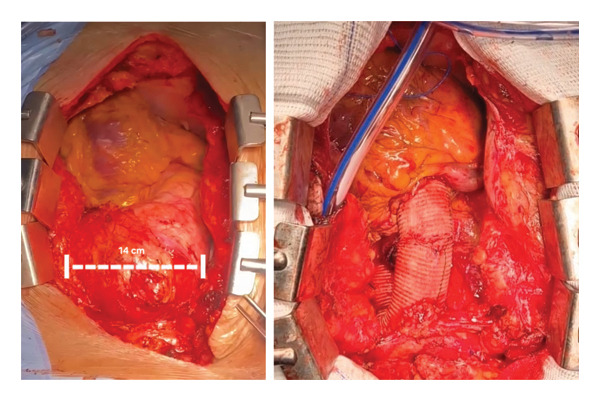
Intraoperative images before and after surgical correction of the aneurysm.

The procedure was performed via median sternotomy. The ascending aorta and entire aortic arch were replaced using a 28 mm × 30 cm Dacron graft (Figure 3). Deep hypothermic circulatory arrest (DHCA) was instituted at a core temperature of 20°C, with a total circulatory arrest time of 35 min. Selective antegrade cerebral perfusion (SACP) was delivered via cannulation of the brachiocephalic trunk throughout the arrest period. The supraaortic trunks were reimplanted en bloc into the graft using the island technique. Cardiopulmonary bypass time was 2 h and 23 min, and aortic crossclamp time was 1 h and 55 min.

The postoperative course was complicated by a left pleural effusion requiring therapeutic thoracentesis and by transient delirium with upper limb myoclonus. Neurological evaluation and imaging revealed no structural abnormalities, and symptoms resolved fully with supportive care.

Histopathologic examination demonstrated advanced atherosclerotic changes with dystrophic calcification, along with inflammatory infiltrates affecting the vasa vasorum, consistent with syphilitic aortitis. This mixed pattern suggests coexistence of chronic infectious aortitis with degenerative aortic disease.

At two‐month follow‐up, the patient exhibited complete clinical recovery, with improvement in anemia, resolution of chest discomfort, and full restoration of functional capacity. VDRL titers decreased from 1:32 to 1:8, indicating appropriate serologic response. Continued outpatient surveillance with periodic imaging was planned.

**Table 1:** Comparative literature review.

Study	Segment	Max diameter (mm)	Notes
Bilman et al. [10]	Arch	72	Contained rupture
Malyshev et al. [6]	Ascending	83	Severe AR
Velasco‐Castro et al. [5]	Ascending	65	Young patient
Ip et al. [8]	Ascending/root	58	Coronary ostial stenosis
Roberts et al. [7]	Ascending	55	“Classic pattern”
Present case	Arch predominant	114	Largest modern arch‐dominant case identified

## 3. Discussion

This case highlights the persistent and evolving relevance of CVS in the contemporary era, demonstrating how tertiary manifestations of *T. pallidum* infection can remain clinically silent for decades until discovered incidentally or after life‐threatening complications. Although the overall incidence has declined dramatically since the introduction of penicillin, the last two decades have seen a worldwide resurgence of syphilis driven by complex socioeconomic, behavioral, and structural factors—including increased rates of unprotected sexual activity, disparities in health access, and coinfection with HIV [1, 5]. As a consequence, tertiary syphilis, while still rare, is no longer an exclusively historical phenomenon and continues to challenge clinicians with its protean and often subtle presentations.

The hallmark of CVS is obliterative endarteritis of the vasa vasorum, leading to ischemic injury of the aortic media, fragmentation of elastic fibers, and progressive dilation of the aortic wall [7]. Classical descriptions emphasize involvement of the tubular ascending aorta with relative sparing of the aortic root—an important point in differentiating syphilitic aortitis from other inflammatory or degenerative etiologies [4, 8]. However, the present case diverges from this typical pattern. The aneurysm demonstrated predominant involvement of the aortic arch, an anatomical distribution that is exceptionally uncommon in modern literature and rarely described even in preantibiotic era series. This atypical morphology may reflect a shift in the phenotype of tertiary syphilitic aortitis as it occurs in older individuals with concomitant atherosclerosis, in whom infectious and degenerative processes may act synergistically to accelerate aortic degeneration.

The aneurysm in this case reached extreme dimensions (114 × 94 × 93 mm), placing it among the largest syphilitic thoracic aneurysms reported in the last four decades. Contemporary literature review reveals that most syphilitic aneurysms today fall within the 55–80 mm range, with only isolated reports exceeding 90 mm. Giant aneurysms above 110 mm are extraordinarily rare and almost never demonstrate arch predominance. The present case therefore occupies a unique position within the spectrum of CVS, reinforcing its novelty and relevance as a teaching case, especially in the context of modern imaging and surgical techniques.

Beyond its unusual size and location, this case illustrates several diagnostic challenges inherent to CVS. The patient’s only symptom was mild exertional dyspnea, a nonspecific clinical feature that could easily be attributed to hypertension, deconditioning, or anemia. The aneurysm was discovered incidentally, during a preoperative evaluation unrelated to cardiovascular complaints, underscoring how CVS may remain clinically silent until aneurysmal dilatation reaches catastrophic dimensions. In many reported cases, the initial manifestation is rupture, dissection, or acute heart failure due to severe aortic regurgitation [10]. Early imaging triggered by subtle clinical clues—murmurs, abnormal chest radiographs, or unexplained dyspnea—remains essential for timely diagnosis.

Serologic evaluation was instrumental in establishing the etiology. The patient exhibited a high VDRL titer (1:32) and positive TPHA, consistent with late latent or tertiary syphilis. Importantly, serology may occasionally be falsely negative in advanced disease due to the prozone effect or waning antibody titers over decades; in such cases, histopathology remains the gold standard for diagnosis [11]. In this patient, histologic findings—plasma cell–rich inflammation of the vasa vasorum and medial degeneration—corroborated the serologic diagnosis and revealed a mixed pathology with superimposed atherosclerosis, reflecting the evolving phenotype of CVS in aging populations.

Surgical repair remains the cornerstone of treatment for large syphilitic aneurysms, regardless of symptomatology. The 2022 ACC/AHA guidelines recommend intervention for ascending/arch aneurysms ≥ 5.5 cm or earlier in cases of rapid expansion or infectious aortitis [14]. Given the extreme diameter of more than 11 cm, the risk of rupture or compression of adjacent mediastinal structures was imminent. The operative strategy—replacement of the ascending aorta and entire arch under DHCA (20°C) with SACP and the island technique reimplantation of the supraaortic trunks—reflects contemporary best practice for minimizing neurological morbidity in complex arch repair. The transient postoperative neurological symptoms observed in this case are consistent with the systemic inflammatory state associated with tertiary syphilis and major aortic surgery, and their complete resolution underscores the adequacy of the perfusion strategy employed.

Another important clinical teaching point is the need for postoperative serologic follow‐up. The decrease in VDRL titers from 1:32 to 1:8 within two months suggests an appropriate therapeutic response. Long‐term monitoring is essential to ensure sustained decline or stabilization of nontreponemal titers, detect reinfection, and identify potential late involvement of other aortic segments [13]. Patients with CVS require lifelong surveillance, even after complete surgical correction, due to the possibility of distal aortic progression or graft anastomotic degeneration.

Finally, this case also underscores broader public health implications. The reemergence of syphilis reflects gaps in screening, prevention, and access to care. Cardiovascular complications—though rare—are severe, resource‐intensive, and preventable with earlier diagnosis. Cases such as this should prompt renewed attention to syphilis control programs, routine screening in high‐risk populations, and clinician awareness of tertiary manifestations Table 1.

## 4. Conclusion

This case demonstrates that CVS, though historically considered a disease of the past, remains clinically relevant and capable of presenting with dramatic, life‐threatening aortic pathology in the modern era. The discovery of a giant, arch‐predominant syphilitic aneurysm in a minimally symptomatic patient highlights the silent and insidious nature of tertiary syphilis. It also expands the contemporary understanding of CVS by illustrating an anatomical pattern—dominant arch involvement—rarely observed in current literature and underrecognized in clinical practice.

The combination of serologic evidence, characteristic histopathology, and mixed inflammatory–degenerative changes underscores the complex interplay between chronic infection and age‐related aortic disease. Early identification of syphilitic aortitis relies on strong clinical suspicion, particularly when imaging reveals nonatherosclerotic aneurysm morphology or unusual aortic distribution. As this case demonstrates, advanced surgical techniques—including DHCA with SACP and island reimplantation—enable safe and effective correction of extensive arch pathology, resulting in excellent functional recovery even in the presence of giant aneurysmal disease.

The successful decline in VDRL titers following therapy further reinforces the necessity of appropriate antimicrobial treatment and structured postoperative serologic monitoring. Given the ongoing global resurgence of syphilis, clinicians should maintain vigilance for CVS, integrate broader epidemiological awareness into differential diagnosis, and advocate for improved screening and prevention strategies.

Ultimately, this case serves as a compelling reminder that “forgotten diseases” can reemerge with significant cardiovascular consequences. Multidisciplinary collaboration, early recognition, timely surgical intervention, and long‐term surveillance remain fundamental pillars for optimizing outcomes in patients with syphilitic aortitis and extensive thoracic aortic involvement.

## Consent

Written informed consent was obtained from the patient for publication of this case report and any accompanying images. A copy of the written consent is available for review by the editor of this journal.

## Disclosure

All authors read and approved the final version of the manuscript.

## Conflicts of Interest

The authors declare no conflicts of interest.

## Author Contributions

Henrique Madureira Da Rocha Coutinho: conception of the study, surgical management, drafting, and critical revision of the manuscript.

José Xavier López: clinical data collection, cardiology evaluation, and contribution to the discussion.

Juan Luis Muñoz Noblecilla: literature review, data analysis, and assistance in manuscript preparation.

Esmeralci Ferreira: histopathological interpretation, supervision, and critical revision of the manuscript.

Joaquim Henrique De Souza Aguiar Coutinho: surgical assistance, data acquisition, and review of the final version of the manuscript.

## Funding

No funding was received for this manuscript.

## Data Availability

The data that support the findings of this study are available from the corresponding author upon reasonable request.
